# Quantifying the demographic cost of human-related mortality to a raptor population

**DOI:** 10.1371/journal.pone.0172232

**Published:** 2017-02-24

**Authors:** W. Grainger Hunt, J. David Wiens, Peter R. Law, Mark R. Fuller, Teresa L. Hunt, Daniel E. Driscoll, Ronald E. Jackman

**Affiliations:** 1 The Peregrine Fund, Boise, Idaho, United States of America; 2 Predatory Bird Research Group, Long Marine Laboratory, University of California, Santa Cruz, California, United States of America; 3 United States Geological Survey, Forest and Rangeland Ecosystem Science Center, Corvallis, Oregon, United States of America; 4 Centre for African Conservation Ecology, Nelson Mandela Metropolitan University, Port Elizabeth, Republic of South Africa; 5 United States Geological Survey, Forest and Rangeland Ecosystem Science Center, Boise, Idaho, United States of America; 6 Garcia and Associates, San Anselmo, California, United States of America; 7 American Eagle Research Institute, Apache Junction, Arizona, United States of America; University of Lleida, SPAIN

## Abstract

Raptors are exposed to a wide variety of human-related mortality agents, and yet population-level effects are rarely quantified. Doing so requires modeling vital rates in the context of species life-history, behavior, and population dynamics theory. In this paper, we explore the details of such an analysis by focusing on the demography of a resident, tree-nesting population of golden eagles (*Aquila chrysaetos*) in the vicinity of an extensive (142 km^2^) windfarm in California. During 1994–2000, we tracked the fates of >250 radio-marked individuals of four life-stages and conducted five annual surveys of territory occupancy and reproduction. Collisions with wind turbines accounted for 41% of 88 uncensored fatalities, most of which were subadults and nonbreeding adults (floaters). A consistent overall male preponderance in the population meant that females were the limiting sex in this territorial, monogamous species. Estimates of potential population growth rate and associated variance indicated a stable breeding population, but one for which any further decrease in vital rates would require immigrant floaters to fill territory vacancies. Occupancy surveys 5 and 13 years later (2005 and 2013) showed that the nesting population remained intact, and no upward trend was apparent in the proportion of subadult eagles as pair members, a condition that would have suggested a deficit of adult replacements. However, the number of golden eagle pairs required to support windfarm mortality was large. We estimated that the entire annual reproductive output of 216–255 breeding pairs would have been necessary to support published estimates of 55–65 turbine blade-strike fatalities per year. Although the vital rates forming the basis for these calculations may have changed since the data were collected, our approach should be useful for gaining a clearer understanding of how anthropogenic mortality affects the health of raptor populations, particularly those species with delayed maturity and naturally low reproductive rates.

## Introduction

Despite an increased regard for raptors over much of the world this past century as reflected in the many laws protecting them, there exists a largely unmitigated suite of lethal agents to which many raptor species remain chronically exposed. Prominent among them are electrocution [1,2], pesticide exposure [3,4,5], wire collisions [6,7], vehicular strikes [8,9], lead poisoning [10,11], and now, wind turbine blade-strikes [12,13]. Were the aggregate of such factors and events less prevalent, deaths might conceivably be compensated by corresponding reductions in density feedback upon vital rates. But what is known of the overall incidence of raptor mortality in the industrialized world suggests that some populations are reduced to the point where all deaths are additive [14], a prudent assumption for purposes of conservation, and one we examine in this paper.

Estimating the finite rate of population change (λ) is a standard approach to analysis where vital rates, estimated within a study area, can be expected to apply to an entire population. That assumption is less appropriate where the risk of mortality declines with distance from a spatially localized hazard, as exemplified in the current status of wind power deployment in the United States where discrete arrays of turbines exist within the more extensive ranges of raptor populations. As an example, we consider a resident, tree-nesting population of golden eagles (*Aquila chrysaetos*) in the vicinity of the Altamont Pass Wind Resource Area (hereafter "windfarm") in west-central California, USA. Turbine construction began there in 1982, and by 1987, about 6,500 wind turbines had been distributed over 16,000 hectares. Soon thereafter, wildlife agencies began receiving reports of raptors killed by turbine blade-strikes, the most frequently encountered being red-tailed hawks (*Buteo jamaicensis*), American kestrels (*Falco sparverius*), golden eagles [15], and (later) burrowing owls (*Athene cunicularia*) [16]. Extrapolating from foot surveys conducted along the rows of turbines in the early 1990s, Orloff and Flannery [15] estimated that about 40 golden eagles died from collisions with wind turbines in the Altamont Pass windfarm each year. Later estimates, based on facility-wide extrapolations during 1998–2007, ranged from 55 to 65 golden eagle fatalities per year [17,18].

Mortality at this level suggested the possibility of population-scale impact upon golden eagles in the region. During an intensive radio-telemetry study from 1994–2000, we gathered field data on survival and reproduction, followed by periodic surveys of territory occupancy and breeder-age-class distribution, through 2014. We presented some of our results in a series of federal and state agency reports [19–21] and here provide a more detailed assessment of the overall cost of anthropogenic mortality to the local population of golden eagles for the period of study. In doing so, we make use of an equilibrium model built in part upon life-history phenomena underlying the population dynamics of this and other territorial, monogamous raptor species. We anticipate that our approach may be useful in other such assessments, given the proliferation of anthropogenic hazards to raptors worldwide.

## Materials and methods

### Study area

**Fig 1:**
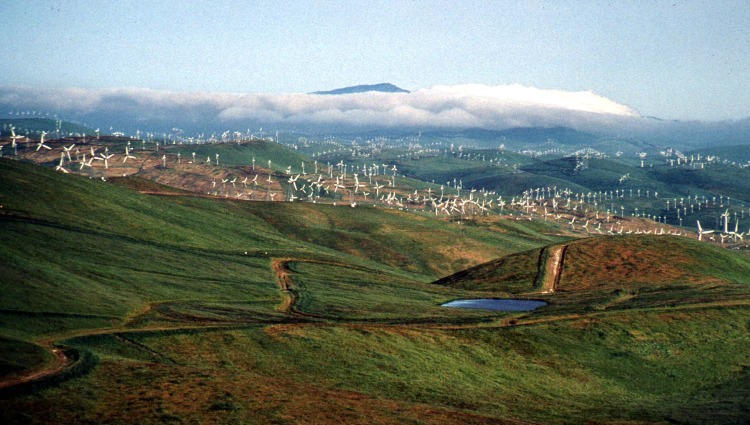
The Altamont Pass windfarm as viewed from its southern boundary in April 1995. The dense configurations of small turbines in this photo are currently being replaced by fewer, larger, and more widely spaced machines.

Altamont Pass lies just east of the metropolitan area surrounding San Francisco Bay in west-central California. The climate is Mediterranean with cool, wet winters and hot, dry summers; average annual precipitation is 389 mm. Strong winds are drawn through the pass from the ocean to the warmer Central Valley, especially in summer. Most of the 142-km^2^ windfarm lies within privately owned cattle ranches in hilly grassland (elevation 60–550 m) dominated by European annual grasses, and with occasional oaks (*Quercus* spp.), eucalyptus (*Eucalyptus* spp.), and California buckeye (*Aesculus californica*) (**Fig 1**). The Altamont Pass windfarm is situated within the Diablo Mountain Range extending north and south. The terrain flattens along the eastern edge of the windfarm, giving way to the continuous farmland of the California Central Valley; urban sprawl lies just beyond the hilly western boundary. During the period of radio-tracking (1994–2000), the windfarm included approximately 4,930 operational wind turbines with a total rated capacity of 580 MW [16]. The most common variety was the relatively small and now obsolete "Kenetech 56–100," usually arranged in tight rows.

**Fig 2:**
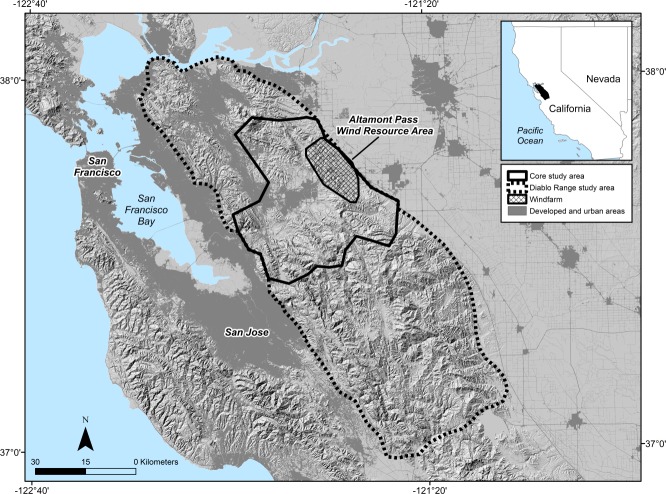
**The Diablo Range golden eagle study region of west-central California showing the location of the Altamont Pass Wind Resource Area (hatched area) relative to: 1) the core study area where we monitored territory occupancy and reproduction of golden eagles, and 2) the broader Diablo Range study area where we used aircraft to regularly monitor movements and survival of radio-tagged eagles during 1994**–**2000, and where Wiens et al. [22] conducted occupancy surveys in 2014 and 2015.**

We conducted surveys of breeding territory occupancy and reproduction within 30 km of the windfarm and referred to this ca. 1500-km^2^ portion as the core study area. We also established a 5,560-km^2^ study area in the broader Diablo Range that encompassed the core study area and was used to conduct aerial monitoring surveys of radio-marked eagles (**Fig 2**). The Diablo Range study area (DR study area) also served as the basis for occupancy surveys later used to estimate the total number of territorial pairs within it [22]. It is bounded on the north by the Sacramento River delta, on the east by the San Joaquin Valley, on the west by the cities along San Francisco Bay, and on the south by State Highway 152 between the towns of Morgan Hill and Los Banos (**Fig 2**). Terrain varies from 0 to 1,333 m above sea level. This pastoral region of the Diablo Range, with several peaks >1000 m, supports open grasslands, oak savanna, oak woodland, pine-oak woodland, chaparral/scrub, and contains a band of urban communities extending between the towns of Livermore and Concord.

### Study species and population

**Fig 3:**
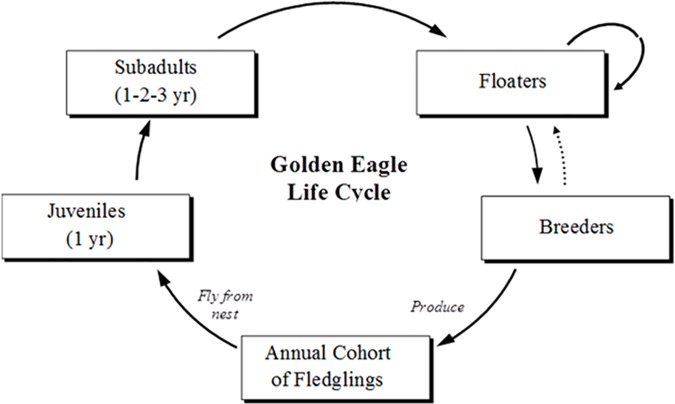
Golden eagle life cycle.

The life cycle of golden eagles includes four post-fledging life-stages, including juveniles (from fledging to one year after fledging), subadults (for 3 years), floaters (non-breeding adults), and breeders (nesting territory holders) (**Fig 3**). Populations of golden eagles are known for their stability in breeding numbers [23,24]. In dry regions, pair distribution usually corresponds to a scattering of cliffs suitable for nesting, but where nesting habitat is continuous, pairs may partition the landscape into a mosaic of contiguous breeding territories from which other eagles are excluded [23]. In either case, breeding territory saturation can be expected to produce a contingent of nonbreeding adults (floaters) that buffer the breeding segment of a population against decline by filling territory vacancies as they occur (see **Fig 3**). This process of cohort limitation and buffering holds population numbers within a range of values known as Moffat's equilibrium [25,26]. Territorial incursions by floaters may modulate population size by interfering with nesting activities, and floaters may usurp territories by evicting or killing breeders [27].

**Fig 4:**
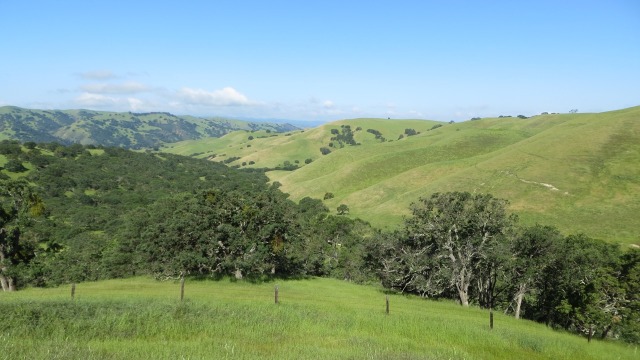
High quality nesting habitat for golden eagles in the Diablo Range, California.

Golden eagles in our core study area nest almost exclusively in oak savanna, oak woodland, and pine-oak woodland (**Fig 4**). Territories in this area are generally contiguous, with pairs defending them year-round and mainly foraging within them. Terrain features tend to shield nesting locations from the view of neighboring pairs. Eagles in the Diablo Range begin nesting in January, lay 1–3 eggs in mid-to-late February (peak period), and fledge their 10–11-week-old young in mid-June. Fledglings usually stay within their natal territories until September. The vast majority of nests are in trees, but a few pairs nest on cliffs, or on electrical transmission towers traversing grasslands where natural structures are unavailable. California ground squirrels (*Otospermophilus beecheyi*) are the principal prey of golden eagles in the region and are widespread and numerous except in areas where their numbers are controlled with summer applications of anticoagulant rodenticides. Other important prey include black-tailed jackrabbits (*Lepus californicus*), cottontail rabbits (*Sylvilagus audubonii*), and black-tailed deer (*Odocoileus hemionus*).

Throughout our investigation, the core study area contained an extraordinary density of golden eagle breeding territories. In 2014, for example, we observed 56 territorial pairs in an 852-km^2^ rectangle south of the windfarm (67.7 pairs per 1,000 km^2^ or 15.2 km^2^ per pair). We estimated eight additional pairs in unsurveyed areas of that space, and if accurate, the average territory would have been 13.3 km^2^ in size (75.2 pairs per 1000 km^2^). Based on an expanded survey effort in 2014, Wiens et al. [22] estimated that the 5,168-km^2^ of non-urbanized terrain in the DR study area contained approximately 280 territorial pairs of golden eagles (18.5 km^2^ per pair). Overall, these are much greater densities than elsewhere reported for this species (see [23]), and the largely contiguous configuration of territories throughout our survey areas suggested saturation by eagle pairs across much of the landscape.

### Population sampling

Estimates of vital rates underlying our demographic analyses derived from data we obtained during the course of our field study (1994–2000), and techniques for their acquisition are described in **S1 Appendix**. The work included surveys of golden eagle territory occupancy and reproduction, eagle capture, radio-marking, aging, sexing, radio-monitoring, and recovery of fatalities of radio-marked birds for necropsy. Our study did not involve surveying the windfarm for fatalities of unmarked birds, so we relied instead on published estimates.

#### Territory occupancy and reproduction

We conducted nine annual surveys of nesting pairs from 1994–2013, concentrating our effort upon a sample of 59–69 territories mostly within 30 km of the windfarm boundary. The first two survey years (1994, 1995) were directed at locating a sample of territories. Five subsequent surveys (1996–2000) met the criteria for reducing bias in estimates of reproductive rate as described by Steenhof [28] as pertaining to failed pairs being more difficult to detect late in the breeding season. Reproductive rate was expressed as the number of ca. 7.5-week-old female fledglings per territorial pair. Surveys in 2005 and 2013 focused on breeding territory occupancy rate and frequency of subadult pair-members as indicators of recruitment potential [29] for comparisons with earlier surveys.

#### Radio-marking

During 1994–1999, we radio-tagged 257 golden eagles representing four life-stages, including 132 juveniles, 64 subadults, 21 floaters, and 41 paired, territorial adults (hereafter "breeders," **Fig 3**); transmitters had expected 4-year battery lives and mortality sensors (see **S1 Appendix**). We tagged 51% of our sample within 10 km of the windfarm, 92% within 30 km, and all within 43 km. The juvenile sample included 101 individuals tagged as 8–9-wk-old fledglings at nests, and 31 as free-ranging birds. For demographic analysis, we considered eagles from fledging to one year after fledging (standardized to 15 June) as juveniles. Three subsequent years of subadulthood included: subadult-1 (13–24 months after fledging), subadult-2 (25–36 months), and subadult-3 (37–48 months) (**Fig 3**).We classified radio-tagged eagles as adults when they reached 15 June of their fifth calendar year (≥48 months after fledging).

#### Radio-monitoring

Weather permitting, we performed aerial searches of the entire DR study area (**Fig 2**) by fixed-wing aircraft at least twice per month from January 1994 through December 1997 (182 surveys), at least once every two months in 1998 (7 surveys), and thereafter at least once per month through September 2000 (43 surveys). Each survey typically required 6–8 hours to complete. We conducted an additional 40 surveys in the windfarm vicinity and 14 surveys outside the DR study area (up to 350 km from the windfarm) to search for missing birds. We compared the proportional occurrence of the various golden eagle life-stages in the windfarm by tabulating the presence or absence of each individual at least once per month during aerial surveys. We recovered fatalities as soon as possible and recorded information pertaining to the cause of death. In cases where the cause was not obvious in the field, we submitted eagle carcasses for necropsy to the California Department of Fish and Wildlife, the U.S. Fish and Wildlife Service, and several private veterinarians.

### Parameterizing the demographic models

#### Survival

We estimated survival probabilities among each of the four life-stages of radio-marked golden eagles: juveniles, subadults, floaters, and breeders. Sample sizes representing the older life-stages increased over the course of the study as eagles recruited from one segment of the population to another. The sample of radio-marked individuals used for our analysis of survival thus included 101 juveniles radio-tagged as fledglings at the nest and monitored for a full year, 155 subadults, 51 floaters, and 47 breeders.

The objective of our analysis was to estimate stage- and gender-specific survival probabilities of golden eagles over seasonal (3-mo) and annual (12-mo) time intervals. Seasonal intervals were: Winter (Dec–Feb), Spring (Mar–May), Summer (Jun–Aug), and Fall (Sep–Nov). Sample sizes for each stage-class gradually diminished over time as radio-tagged eagles died or were censored because of transmitter failure or unexplained signal loss. As a consequence, we did not estimate survival for seasonal intervals in which sample sizes fell below 11 individuals within a given stage-class. This resulted in truncating survival to 12 seasonal (3-mo) intervals for subadults and floaters, and 16 for breeders. We assumed that fates were independent among each member of two breeding pairs and among juvenile eagles radio-tagged as siblings (N = 28 broods). We assumed that four unrecoverable, stationary transmitters emitting mortality signals were fatalities, and not prematurely detached. We based that decision on data showing that six of seven recoveries of detached transmitters involved birds tagged in the initial 6 months of the study when the transmitter attachment procedure was undergoing refinement; the birds in question were not part of that initial sample. We estimated annual survival (*S*) of radio-marked eagles with known-fate models in Program MARK [30] which allowed for staggered entry of individuals into the analysis and censoring of those that left the DR study area or could not be relocated [31].

Known-fate parameter estimation in Program MARK uses a modification to the risk set [32] in which animals are included in an interval only when they are relocated. Although uncertain relocation (i.e., data censoring) results in a loss of precision of the estimate, the modified estimator remains relatively unbiased as long as data censoring is independent of fate [33]. Data censoring would be expected in our study if radio-tagged eagles had dispersed beyond our aerial survey area, or if a radio had prematurely malfunctioned, or was destroyed by the lethal agent, for example, by a wind turbine blade. To evaluate the effect of reducing various types of mortality on estimates of population trend (see below), we estimated survival in three ways: 1) with all observed deaths included, 2) with cases of all turbine-related deaths censored, and 3) with all deaths known to be human-related censored.

We conducted separate analyses in Program MARK to estimate survival for each life-stage because some individuals tracked for more than 1 year could contribute to survival estimates of more than one age-class, and we wanted to maintain independence among stage-specific estimates of survival. We considered a limited set of four *a priori* candidate models to examine potential variation in survival among seasons (i.e., 3-mo time intervals) and between sexes for each stage-class:

1. Survival is constant over seasons (3-mo time intervals), {*S*(.)}
2. Survival varies among seasons, {*S*(t)}
3. Survival is dependent on sex, {*S*(sex)}
4. Interactive effect of season and sex on survival, {*S*(sex × t)}

We ranked the four candidate models using the second-order Akaike’s Information Criterion for small sample sizes (AIC_*C*_), and evaluated the strength of evidence for each model with ΔAIC_*c*_ (i.e., the difference between the lowest AIC_*C*_ value and the AIC_*C*_ from all other models), Akaike weights, and evidence ratios [34]. We obtained estimates of annual survival for each age-class using seasonal survival estimates from Program MARK, and approximated the variance of annual survival in such cases using the delta method [35].

#### Fecundity

We estimated reproductive rates for both one- and two-sex models. We based the population rate-of-change estimate (see below) on the number of female fledglings per territorial pair, an informed decision underscoring the importance of obtaining sex-ratio data in trend studies of monogamous species [36]. The consistent male bias we found among fledglings, free-ranging eagles, and fatalities meant that fewer females than males would have been available to replace dead breeders. Under a scenario of floater depletion, females would be lost first, and because reproduction requires active participation by one male and one female in this species, females were the limiting sex.

We estimated the reproductive rate by first averaging the annual number of ca. 7.5-week-old fledglings per territorial pair, and then multiplying by the average proportion of female fledglings we encountered overall. We calculated the standard error of the reproductive estimate by the delta method applied to the product of the two variables.

### Population modeling

#### Population rate-of-change

We defined the potential growth rate (λ_p_) as that shown by a hypothetical population in which all female eagles obtain breeding territories and reproduce at the average rate during their first year of adulthood which, according to our protocol, begins 48 months after fledging (mid-June). Their fledged young, however, cannot materialize for another 12 months, meaning that the adult parent of incipient fledglings is, itself, 60 months post-fledging. For the growth computation, we employed a standard, single-sex, post-reproductive pulse matrix model [37] that included the juvenile year, three successive years of subadulthood, and breeders, while ignoring the possibility of floaters as a life-stage. In an alternative scenario, conceivable under these same conditions of no competition for territory ownership (and no other density dependence), we modeled the outcome with the assumption that females become breeders a year earlier, namely as third-year subadults; **S1 Appendix** provides computational details.

#### Floater-to-breeder ratio

To compute an estimate of the floater-to-breeder ratio at Moffat's equilibrium [26], we extended the matrix models of **S2 Appendix** to include floaters as a life-stage that begins when subadults transition to adulthood four years after fledging. Imposing equilibrium on this model allows one to extract the floater-to-breeder ratio, as explained in **S3 Appendix**.

#### Demographic cost of mortality

We quantified the demographic cost of turbine-induced mortality by calculating the number of breeding pairs just required to sustain themselves and the annual rate of blade-strike mortality. We based our assessment on estimates of vital rates, the death toll, and the average age of blade-strike death in months. For the latter, we used mortality data from juveniles and subadults radio-tagged as fledglings, then added the proportion of adults among eagles found by wind industry workers during 1989–1999. Because the age of adults beyond the fifth calendar year of life could not be ascertained, we regarded each adult fatality as a first-year adult under the assumption that an eagle of that age would reproduce at the average rate [38]. We developed two simple (two-sex) models in **S4 Appendix**, the first providing a direct cost estimate from the results of this study, and the second requiring more precise knowledge of the age of each subadult fatality. Both models focused on the demographic cost of one fatality which could then be scaled linearly with estimates of the annual number of blade-strike deaths.

### Ethical approval

Field work was performed under an animal use protocol approved by the Institutional Animal Care and Use Committee of the University of California, Santa Cruz, which is registered as a research institution by the U.S. Department of Agriculture (Research Number R-4029). Permitting agencies included the California Department of Fish and Wildlife, the U.S. Geological Survey Bird Banding Laboratory (Permit #20675), and the U.S. Fish and Wildlife Service. All land access was by permission from owners or land managers.

## Results

### Distribution of radio-tagged eagles

The aerial surveys and corresponding movements data showed that the majority of golden eagles we radio-tagged could be considered residents of the DR study area (**Fig 2**). Eagles tagged as fledglings tended to remain year-round, although a few departed, then returned. Among the 117 individuals radio-tagged as free-ranging nonbreeders (captured primarily in winter), 108 survived long enough to suggest their geographic affiliation. Ninety (83%) were regularly detected within the DR study area through the summer. Movements of 7 others (6%) suggested residency in the larger region of west-central California. Eleven (10%) were detected only in winter and spring, and so may have originated elsewhere, although some among them may have remained undetected with failed transmitters. Two of 49 deaths among non-breeders captured and marked as free-ranging individuals were located outside the DR study area; one collided with a wind turbine at another windfarm ca. 30 km north of the study area, and the other died ca. 110 km east near the town of Coulterville, California. Juveniles, subadults, and floaters wandered throughout the DR study area, but aggregated in its northern portion, especially in the vicinity of the windfarm.

**Fig 5:**
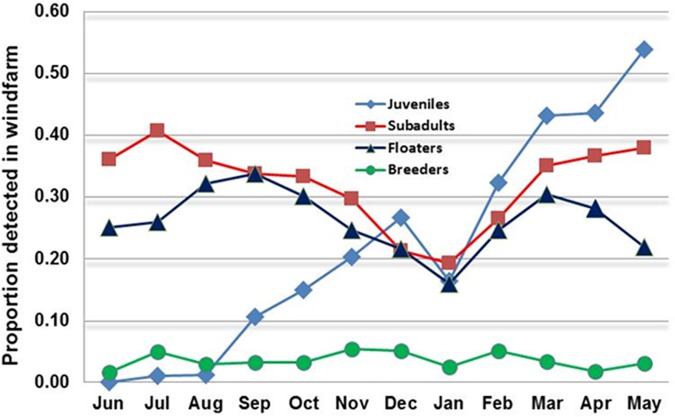
Monthly variation in the proportion of individual golden eagles detected in the windfarm at least once per month in aerial surveys of 101 juveniles (radio-tagged as fledglings), 155 subadults, 51 floaters, and 47 breeders in the Diablo Range study area, 1994–2000. The increase in percentage of juvenile occurrence in the windfarm resulted as eagles radio-tagged as fledglings in May and June dispersed from their natal territories during September–November of each year.

We found that breeders generally remained in or near their territories year-round and only occasionally entered the windfarm. In contrast, we detected subadults and floaters far more frequently in the windfarm and, whereas juveniles tended to remain in the vicinities of their natal territories until at least September, they later appeared in the windfarm in proportions comparable to those of subadults and floaters (**Fig 5, S5 Appendix**).

### Evidence of floaters

We radio-monitored 51 floaters for varying periods before death or censoring, identifying them as adults whose movements continually indicated the lack of a territory. We tagged 19 as adults and tracked them for 2–43 months (median = 19 months) prior to death (n = 11) or censoring; none became territory holders during monitoring. One adult—possibly a winter migrant breeding elsewhere—was absent from the DR study area in spring and summer of three consecutive years (1994–1996), and eventually died from a turbine blade-strike. Fourteen floaters tagged as third-year subadults were monitored for 4–52 months (median = 21); seven died (1 outside the study area) and two obtained territories. Radio-life (ca. 4 years, see **S1 Appendix**) likely limited the recorded tenure of 17 other floaters tagged as younger subadults and fledglings (range as floaters = 1–45 months, median = 9 months); four died. One male, tagged as a breeder in May of 1996, remained as such until being replaced in its territory by another male in February 1998; the deposed male floated for 11 months before dying of unknown causes within a territory occupied by an established pair. In all, the 51 breeding-age eagles floated for a total of 959 monitoring months in the study area, and only two acquired breeding territories.

### Sex ratio

A consistent male bias characterized all age groups of the studied population. Fledgling eagles encountered during the four years of radio-tagging (1994, 1995, 1996, and 1999) showed male-to-female ratios of 18:13, 13:9, 16:9, and 21:8, the aggregate yielding 64% males, a significant departure from 1:1 (G-test; G = 7.96, p = 0.005). Samples of free-ranging, non-territorial eagles captured for radio-tagging during 1994–1999 contained 62% males (76:47 individuals), again a significant departure from parity (G = 6.90, p = 0.009) and suggesting no post-fledging sex-bias in mortality. Indeed, 88 uncensored fatalities showed 64% males, and a larger sample of 131 fatalities, both censored and uncensored, including band recoveries after battery failure through 2009, contained 64% males. Brood analysis suggested that the ratios existed at hatching and did not result from female-biased nestling mortality, as follows. Assuming that all pairs laid two, or rarely three, eggs, 38 nests with single fledglings contained 23 males and 15 females. Among 32 broods of 2 fledglings, 4 contained 2 females, 14 had 2 males, and 12 consisted of a male and a female. Three 3-fledgling broods contained 5 males and 4 females. Overall sex ratios between nests with one fledgling (61% males) and those containing two or more (62%) were virtually identical.

### Fecundity

Five surveys of reproduction during 1996–2000 focused upon 59–69 territories (**Table 1**). Overall average reproduction for the 5 years was 0.638 fledglings per occupied territory. Applying the estimate of fledgling sex ratio (0.64 males) yielded 0.2313 (SE = 0.040) female fledglings per female territory-holder. As observed elsewhere [39], nest success, as largely influenced by failure to lay eggs, accounted almost entirely for annual differences in productivity, whereas mean brood-size showed comparatively little variation among years (**Table 1).**

**Table 1:** Results of golden eagle nest surveys in the Diablo Range study area, 1996–2000.

	1996	1997	1998	1999	2000
Pairs surveyed	59	59	64	69	67
Fledglings	39	35	37	62	31
Fledglings per pair	0.66	0.59	0.58	0.90	0.46
Fledgling broods	27	22	29	40	22
Mean brood size	1.44	1.59	1.28	1.55	1.41
Nest success rate	0.46	0.37	0.45	0.58	0.33

### Territory occupancy and breeder age

In 2005 and 2013, as an empirical test of population stability, we resurveyed 58 territories that had been occupied by pairs in 2000. All contained pairs in 2005, and all but two contained pairs in 2013. Circumstantial factors explained one of the two vacancies in 2013, but the other remained unresolved. We detected no significant upward trend in the proportion of subadults as pair members over the years as might have been expected if the population had declined to the point of losing its floater buffer (**Table 2**). Interestingly, 13 of the 15 subadults were females, and among 10 subadult females whose reproductive outcome was known, three (30%) fledged young.

**Table 2:** Ages of breeding golden eagles at territories within 30 km of the Altamont windfarm. The asterisk indicates that the calculation included two individuals of uncertain age and therefore gave the maximum possible representation of subadults for that year. Note that yearly variation in the number of aged eagles reflects differences in sampling effort rather than population.

Male	Female	1996	1997	1998	1999	2000	2005	2013
adult	adult	48	41	49	54	55	54	51
subadult	adult	0	0	0	1	0	0	1
adult	subadult	2	0	2	3	2	1	3
adult	age uncertain	0	0	0	0	0	1	0
age uncertain	adult	0	0	0	0	0	1	0
	Birds aged	100	82	102	116	114	112	110
	Percent subadults	2.0	0.0	2.0	3.4	1.8	2.6*	3.6

### Fatalities

At least 59 (67%) of 88 uncensored fatalities were human-related, and additional human-related deaths likely existed within the subset of 18 undiagnosed fatalities, eight of which were recovered intact, showing no trauma, meaning that some may have been poisoned (**Table 3**). Eleven deaths (12.5%) were determined to have been of natural causes, including 6 recently-fledged juveniles, one breeder that died of botulism, and four adults (two breeders and two floaters) that died from territorial encounters with other eagles. Circumstantial evidence suggested that two additional deaths among undiagnosed breeder and floater fatalities may have resulted from territorial fighting. One undiagnosed fatality, a juvenile 2 months post-fledging, was emaciated, suggesting foraging incompetence, or perhaps lead poisoning, a frequent manifestation of which is emaciation from peristaltic paralysis [40]. No other evidence implicated starvation as a cause of death among free-ranging eagles except in cases where blade-strikes had rendered them flightless. Note that, in our vital rates analyses, we treated all 18 undiagnosed deaths as "natural" so as to obtain a minimum estimate of anthropogenic mortality and a maximum estimate of natural mortality (**Table 3**).

**Table 3:** Causes of death among 88 uncensored golden eagle fatalities during 1994–2000. Juveniles include only those radio-tagged as fledglings. Undiagnosed fatalities included four unrecovered individuals (see Methods).

Mortality agent	Juveniles	Subadults	Floaters	Breeders	Total	Percent	Anthro-
	(n = 101)	(n = 155)	(n = 51)	(n = 47)	Fatalities	of total	pogenic
**Wind turbine blade-strike**	0	28	6	2	36	40.9%	Yes
**Undiagnosed fatality**	4	4	5	5	18	20.5%	?
**Electrocution**	4	5	2	0	11	12.5%	Yes
**Fledgling mishap**	6	0	0	0	6	6.8%	No
**Killed by eagle**	0	0	2	2	4	4.5%	No
**Wire strike**	1	2	1	0	4	4.5%	Yes
**Vehicular strike**	0	2	1	0	3	3.4%	Yes
**Lead**	0	2	0	1	3	3.4%	Yes
**Botulism**	0	0	0	1	1	1.1%	No
**Brodifacoum poisoning**	0	0	0	1	1	1.1%	Yes
**Gunshot**	0	0	1	0	1	1.1%	Yes
	**15**	**43**	**18**	**12**	**88**	**100%**	**≥59**

Wind turbine blades killed 36 (40.9%) of the 88 uncensored fatalities (**Table 3**), followed by 11 electrocutions (12.5%). Among the blade-strike fatalities, 38.9% occurred in the spring months (March-May), 30.6% in summer, 16.7% in winter, and 13.9% in fall. All electrocutions involved utility lines, and four or possibly five were actually wire strikes of eagles electrocuted as they contacted closely spaced conductor wires at mid-span between utility poles; in one direct observation, a gliding adult (untagged) was electrocuted as it descended vertically between two wires.

Only two (16.7%) of 12 uncensored fatalities recorded among the 47 radio-tagged breeders were caused by turbines. We found no turbine blade-strike fatalities among the 101 juveniles radio-tagged as fledglings, and only one turbine death among the 31 juveniles tagged as free-ranging individuals, that single fatality occurring in the last month of the juvenile year. In contrast, radio-tagged subadults and floaters were highly vulnerable to turbine blades (**S5 Appendix**). Twenty-eight of 36 uncensored blade-strike fatalities occurred within our sample of 155 subadults, and six among 51 floaters (**Table 3**). The numbers of blade-strike deaths among some cohorts were substantial. We tagged 25 fledgling eagles in 1994, and a year later, six of these had died (none from turbines) or disappeared, leaving 19 in the DR study area as first-year subadults. From January 1995 to November 1999, turbine blades killed at least 11 of these eagles (including censored ones), an attrition of at least 58% arising from this single mortality agent. Only one was known to have died of other causes within the study area during this period. Of 16 radio-tagged eagles from the 1995 cohort detected in the study area as subadults, six (37.5%) were eventually recorded as killed by wind turbines (March 1997 –May 1999). We recorded five blade-strike deaths among 13 subadults and floaters remaining in the study area from the 1996 cohort, a kill rate of 38 percent. We tracked the 1999 cohort through only their first summer of subadulthood, and among 19 of these eagles detected in the study area as subadults, four (21.0%) had been killed by turbine blades when radio-monitoring was concluded in September 2000. Note that all these figures on turbine-related mortality represent minimum incidence because the blades may have destroyed a proportion of transmitters.

Eagles fledging from nests near the windfarm appeared no more likely to be killed there than those originating from more distant sites within our sample. Our results showed no difference in median or mean distance from natal site to the windfarm between those killed by turbines and those that were not. The median distance from the natal site to the windfarm for 22 turbine-killed subadults and floaters was 11.3 km (mean = 13.2, SD = 9.1), while the median for 38 such eagles not killed by wind turbines was 11.7 km (mean = 13.3, SD = 9.1).

### Survival

Annual survival probabilities, with all known (uncensored) deaths, ranged from 0.801 for subadults to 0.905 for breeders (**Table 4**). The relative importance of human-related mortality varied substantially among the four stage-classes, as shown by differences between estimates of survival with and without turbine- or other known human-caused deaths included. This difference was greatest for subadults (0.18), followed by floaters (0.09), juveniles (0.05), then breeders (0.03). Based on model selection results, we found evidence for a peak in juvenile mortality just after fledging ("fledgling mishaps," **Table 4**), but no indication of seasonal variation in survival of subadults, breeders, or floaters. We found little evidence for a sex-dependent effect on survival (**S6 Appendix**).

**Table 4:** Probability of annual survival ($\hat{S}$) for four stage-classes of golden eagles radio-marked in the vicinity of the Altamont Pass Wind Resource Area, California, 1994–2000. We show survival estimates with and without turbine-related and known human-caused deaths included in the analysis. All undiagnosed deaths were treated as "natural" so as to obtain a maximum estimate of natural mortality (see text). No juveniles were killed by turbine blade-strikes.

Stage-class	Number of individuals included in estimate (Females, Males)	Annual survival probability ($\hat{S}$)	$\hat{SE}$	95% confidence interval	
				Lower	Upper
Juveniles					
All deaths	101 (35, 66)	0.842	0.038	0.753	0.903
Turbine-related deaths censored	101 (35, 66)	0.842	0.038	0.753	0.903
Human-caused deaths censored	98 (33, 65)	0.893	0.032	0.812	0.942
Subadults					
All deaths	155 (61, 94)	0.801	0.028	0.747	0.856
Turbine-related deaths censored	150 (58, 92)	0.921	0.020	0.882	0.959
Human-caused deaths censored	147 (56, 91)	0.978	0.011	0.957	0.999
Floaters					
All deaths	51 (17, 34)	0.839	0.040	0.761	0.916
Turbine-related deaths censored	51 (17, 34)	0.870	0.037	0.799	0.942
Human-caused deaths censored	50 (17, 33)	0.924	0.030	0.866	0.983
Breeders					
All deaths	47 (29, 18)	0.905	0.026	0.853	0.956
Turbine-related deaths censored	47 (29, 18)	0.920	0.024	0.872	0.967
Human-caused deaths censored	47 (29, 18)	0.935	0.022	0.892	0.979

### Potential population growth rate

Assuming that the minimum age of a post-reproductive adult female was 60 months, we estimated λ_p_ = 0.997 (SE = 0.025). In the case of third-year subadults breeding in the absence of competition with older eagles, as implicit in the growth model (see **S2 Appendix**), λ_p_ = 1.003 (SE = 0.026). These results describe a population that is neither increasing nor decreasing (λ~1.0), but one for which no floaters are generated beyond those required to fill territory vacancies as they arise.

### Demographic cost

Model 1 in S4 Appendix predicted that the entire annual reproductive output of 3.931 territorial pairs was necessary to supply a single fatality of age 40 months post-fledging, the estimated average age of blade-strike death, and with the assumption that all adult fatalities were first-year adults (see Methods).

## Discussion

Surveys in 2005 and 2013 of a sample of 58 breeding territories in the core study area, all occupied by pairs in 2000, showed that almost no change had occurred in the rate of pair occupancy. Moreover, the proportion of subadult pair members displayed no significant trend of increase that might suggest a deficiency of adult recruits (see [29,39]). These findings of stability support the idea that breeder attrition in the DR study area was regularly buffered by floaters, some internally generated and others likely as immigrants. The most logical source of the latter would have been the continuous and reputedly robust breeding population of golden eagles extending southward from the DR study area through the Southern Coast Ranges and Transverse Ranges of California, then turning northward along the western foothills of the Sierra Nevada. During our surveys for missing radio-marked birds, we occasionally found them within that southern region but only rarely northward or eastward. The movements of 51 radio-marked adults confirmed their identity as floaters, and yet we found that only two acquired territories during periods of up to 52 months of monitoring. We observed very little delay, however, in floater replacement of fatalities detected among radio-marked breeders, and we found occasional evidence of lethal conflict over territory possession. These findings reveal a population that has filled its breeding habitat with territorial pairs right up to the adaptive threshold of site-acceptance where theory predicts advantage to the floater strategy [26,41].

Long-term radio-tracking produced evidence of general, year-round residency and allowed us to quantify mortality, the agents involved, and differences in the types of mortality occurring within the four life-stages (**Table 3**). Subadults and floaters showed a far greater incidence of blade-strike death than juveniles or breeders, the latter tending to remain within their territories outside the boundaries of the windfarm. The virtual immunity of juveniles to blade-strikes during our study was partly explained by their remaining in or near their natal territories during June–September, whereas their presence in the windfarm thereafter became progressively comparable to that of subadults (**Fig 5, S5 Appendix**). A plausible explanation might be that vulnerability to turbine blade-strikes is connected with hunting live prey, an activity in which juveniles are presumably less competent and less participatory than older eagles. In our study area, eagles fledging in mid-June miss the opportunity to exploit the recently emerged crop of vulnerable young ground squirrels, whereas in ensuing years, more experienced subadults learn to hunt them. Doing so usually requires rapid, near-ground maneuvering associated with "contour hunting" [42], often in conditions of high wind-turbulence, with consequent effects upon flight control.

Our radio-tracking data showed a surprisingly low incidence of natural mortality, even despite the defaulting of all 18 undiagnosed deaths to "natural causes" for survival estimation. Diagnosed natural deaths included six juveniles that died in post-fledging mishaps, two of which sustained trauma, and two others starved in dense vegetation, inaccessible to their parents. Only three subadults may have died from natural causes, again, all within the ambiguous subset of undiagnosed fatalities. Two of seven floater deaths were diagnosed as "killed by eagle," and the fates of three others were so suspected, all five having died within golden eagle territories between 13 February and 1 March, the peak period of egg-laying.

Censoring 59 deaths recorded as anthropogenic left 11 determined to be natural and 18 of unknown cause, giving a total of 29 possibly natural fatalities (**Table 3**). Life-stage-specific survival rates calculated from those 29 fatalities ranged from 0.893 for juveniles to 0.978 for subadults (**Table 4**). Again, these are minimum values because some of the 18 undiagnosable fatalities we classified as "natural" were likely of anthropogenic cause. Survival values of such magnitude suggest that pre-industrial golden eagle populations were robust in comparison to what we are able to observe in the modern world, although much would have depended on factors such as prey densities and competitors.

### Population theory

Our demographic analysis is based on the understanding that golden eagle populations develop floating segments rather than show an increase in breeding pairs beyond densities mediated by territorial exclusion [26,27]. The theoretical basis is that, along the continuum of territory quality, there is a threshold of site acceptance, below which, the strategy of rejecting substandard sites and waiting for a better one confers higher fitness than occupying them [26]. The evolutionary stable state defining the quality-threshold of site acceptance (adaptive serviceability) was quantified in oystercatchers (*Haematopus ostralegus*) as that promising replacement-rate reproduction over the lifetime of the tenant [43]. The limits to annual cohort size, resulting from the saturation of adaptively serviceable sites, stabilize floater numbers and therefore population size [25,26].

Floating segments do not develop in territorial bird populations in which low natural reproductive value makes the strategy of waiting for a better territory maladaptive [44,45]. For these populations, and those in which vital rates are depressed or otherwise insufficient to the maintenance of a floating segment, an alternate mode of stabilization may develop, again as a result of adaptive preference for higher-quality territories. Territories producing an excess of offspring (source-sites) provide recruits to otherwise unsustainable sink-sites, and the population stabilizes when recruitment to sink-sites reaches its limit [44,46,47]. This mode of equilibrium has been called the buffer effect [48], source-sink equilibrium [46,49], the habitat heterogeneity hypothesis [50], and site-dependent regulation [51]. We refer to the stable state as site-performance equilibrium (SPE) for reasons explained below. Note that it is immaterial to the equilibrium process whether high-quality sites are scattered or aggregated in discrete patches of similar habitat so long as those sites exist within the normal ranging patterns of prospective occupants [49]. Limits to cohort size underlie Moffat's equilibrium (ME) and SPE, qualifying both as "Moffat models" corresponding to "density levels 2 and 3," respectively, as described by Brown [48].

Golden eagle populations that support floaters of both sexes can be expected to exist at ME, whereas those with chronically depressed vital rates may settle on SPE as numbers fall below that required to fill all territories perceived serviceable by pairs (see [52]). At this point, the population becomes recruitment-limited rather than space-limited. For stabilization to occur, it is necessary that during the decline (or growth from a depressed state), territory-holders gravitate to source sites [51]. The basis for ongoing site-discrimination is, of course, the fitness reward attached to source-site occupancy, and to the extent to which source-sites exist and are favored, the declining eagle population can be expected to restabilize.

Equilibrium numbers at both ME and SPE occur, not as fixed values, but as "clouds" of values over time. In addition to changing environmental influences upon site-quality, the breeding sector contains individuals of varying competence, partly age-related, meaning that a high-quality individual on a low-quality territory may achieve higher reproductive success than expected solely on the basis of site quality, and vice versa [38]. In equilibrium models, individual quality can be thought of as a component of site quality [47,51]. Thus, the "habitat heterogeneity hypothesis" of Dhondt et al. [50] is perhaps better expressed as a "site-performance hypothesis," in that the latter necessarily includes individual reproductive competence as a constituent property [53].

The role of individual quality is, of course, difficult to distinguish from habitat-quality effects, particularly in species with lengthy site-tenure. In a detailed, 16-year study of a population of peregrine falcons (*Falco peregrinus*), however, Zabala and Zuberogoitia [54] found evidence that individual quality was more important than site quality with respect to nest success. Moreover, the authors compellingly argued that, in the population they studied, site-acquisition had almost nothing to do with site quality discrimination, but rather with the vulnerability of weak territory-holders to displacement by floaters. The implication is that genetic variation mediating the capacity for site quality discrimination (above the serviceability threshold) might be somewhat "invisible" to natural selection when a floater buffer is present. From a fitness perspective, competence in site-acquisition and reproduction are sequential components of nest success, and yet only the latter would have significant function in population dynamics (by contributing to cohort size). In populations where ME continues long-term and with a robust floating segment, selection may well favor adaptations relating to success in obtaining and holding a site at the expense of prospering there. Large body size, for example, might confer advantages in male-to-male competition for site-acquisition, yet suboptimize foraging competence once the site is obtained (see [55]).

Food depletion and other modes of density feedback may modulate golden eagle numbers at equilibrium (see [56]), and a notable example is floater pressure upon breeder survival and nest success [27]. Several studies have sought to differentiate between the roles of interference and habitat heterogeneity in explaining density-dependent fecundity [57,58]. In our view, such comparisons require a degree of robustness in the floating segment, given that the tendency of a floater to incur risk in confronting a territory-holder should vary inversely with age-related reproductive value [26]. Thus, thinking of free-ranging juveniles and subadults as potential agents of interference is probably unrealistic [59]. That aside, most such studies have revealed site quality discrimination consistent with the conditions leading to SPE [60], and of note is the work of Sergio et al. [61] where black kites (*Milvus migrans*) returning as migrants sequentially settled on territories of decreasing quality. Among non-territorial species like colonial vultures, on the other hand, one would expect crowding and food competition to be far more regulatory than variation in breeding site quality [62].

### Interpreting lambda

The standard errors of the potential growth estimates almost equally spanned the alternatives of population increase and decrease. If the point estimates (λ~1.0) were true in the study area during 1994–2000, there would have been just enough locally generated recruitment to accommodate the annual demand for replacement of dead breeders. Any further decrease in vital rates would yield a deficit in breeder recruitment unless immigrant floaters were there to fill territory vacancies. Meanwhile, lambda, as a function of vital rates, would at least initially reflect decline by dropping below unity. If, however, in the absence of immigration, any sites endured as sources, and remaining adults gravitated to them, the population might restabilize at SPE with fewer occupied territories, a higher net per-capita fecundity, and lambda returning to unity.

An interesting dynamic beneath the surface of this analysis relates to the time-scale at which human-related mortality has arisen in the study area. Recall that the theoretical basis for the floater option is the rejection of sites perceived to offer less than replacement-rate lifetime reproduction [43]. Depressed survival as a result of anthropogenic mortality means that some proportion of sites formerly offering replacement no longer do so. Eagles prospecting for territories would therefore underestimate the adaptive threshold of site-acceptance until selection adjusted sensitivity to site quality over evolutionary time. If the adjustment was somehow instantaneous with a decline in per-capita survival, however, the number of occupied sites would contract, floaters would appear, and λ_p_ would increase in response to fewer occupied territories from which the reproductive rate was calculated (fledglings per occupied site).

In the event that anthropogenic mortality was to ameliorate, the studied population would generate a floater buffer and ultimately stabilize, with λ_p_ remaining in excess of unity. This is contrary to the occasional misconception that λ_p_ = 1.0 is necessarily consistent with a healthy population [63]. Indeed, any sustained value of λ_p_ > 1.0 denotes ME or progress thereto, whereas λ_p_ = 1 may be a precarious equilibrium, depending on its mode, that is, precarious at ME (for lack of excess floaters) and stable at SPE, being that the occupants of poor quality sites constitute a buffer [48].

As a way of assessing the impact of the blade-strike component of mortality, we censored the windfarm fatalities on the estimated day of death and recalculated survival rates for each life-stage (**Table 4**). Doing so yielded λ_p_ = 1.040 (SE = 0.024), a value that would stabilize the population and produce a floater-to-breeder ratio (F:B) of 0.5 (see **S3 Appendix**) at a moderately buffered ME (see [64]). Going a step further, our minimal approximation of natural survival for each life-stage gave λ_p_ = 1.072 (SE = 0.023) and F:B = 1.5 at ME (**S3 Appendix**). In such a population, the degree to which floater incursions would depress natality and survival among breeders and floaters might well be significant.

At some level of F:B, a substantial change in vital rates (with a necessarily corresponding change in the rate of floater generation) might produce a density-feedback loop regulating the size of the floating segment. Under such conditions, additional deaths might, to some degree, be compensated so long as the feedback dynamic persisted. Note that the latter condition would both modulate ME and strongly buffer the condition of territory saturation. Ironically, the most robust scenario of population resilience might be the situation where floater incursions had the greatest possible negative impact upon reproduction and adult survival (see [65]). Again, as floaters age, they have progressively less residual reproductive value to risk in territorial confrontation and should show an increasing tendency to initiate it [26]. Thus, the effect of floater pressure may not be linear with changes in F:B.

### Demographic cost of windfarm mortality

To assess the direct influence of blade-strike mortality, we estimated the number of golden eagle pairs required to sustain it. The reasoning behind our analysis began with an estimate of the number of pairs necessary to produce a single fatality (**S4 Appendix**). Consider that the observed average number of fledglings (of both sexes) per pair was 0.638 during our study, so the death of a recent fledgling would consume the issue of 1 ÷ 0.638 = 1.567 pairs. We estimated, however, that the average age of blade-strike death during 1987–1997 was 40 months, that is, assuming all adult fatalities were first-year adults (see Methods). Our survival data (with turbine deaths censored) showed the probability of a fledgling surviving 40 months as 0.695, meaning that an eagle of that age was the sole survivor of 1.448 fledglings, the production of which demanded the existence of 2.256 territorial pairs. These pairs were, of course, not self-sustaining in that the 4.512 pair-members each incurred an annual mortality risk of 0.080, thus requiring 4.512 x 0.080 = 0.361 annual replacements (floaters) of at least 56 months of age. We calculated that a 56-month-old eagle is the sole survivor of 1.653 fledglings and therefore 2.590 pairings, meaning that an additional 2.590 x 0.361 = 0.935 pairs were necessary to supply those recruits, yielding a subtotal of 2.256 + 0.935 = 3.190 pairs. Continuing the process through five additional steps leads to 3.844, an approximation of the number of territories supplying each blade-strike death. Model 1 in **S4 Appendix** formalizes this incremental procedure and provides a simple computational formula with result 3.931 for the exact count towards which the previous counts asymptote.

Published estimates of blade-strike deaths occurring during 1998–2007 ranged from about 55 to 65 individuals per year [17]. Thus, if the vital rates we estimated remained valid during that period, the least of those estimates—55 deaths—would have consumed the annual production of 55 x 3.931 = 216 pairs existing at the demographic break-even point, producing no buffer of recruits in excess of that required to sustain themselves. The estimate of 65 annual windfarm deaths reported by Bell and Smallwood [18] would have required the existence of 255 occupied territories.

If one assumes that 90% of the population contributing to windfarm mortality was resident to the DR study area, as the radio-tracking data suggested, and that the likelihood of blade-strike death in the Altamont was a function of natal distance to the windfarm (our data are ambivalent here), then we can estimate a footprint of its influence upon the population in the DR study area. The minimum geographic extent of that influence (90% of 55–65 fatalities) would thus be defined by the distribution of the nearest 195–230 territories. Indeed, the estimated total number of territorial pairs in the DR study area in 2014–2015 was 280 pairs (95% CI = 256–305 pairs) [22], suggesting that the golden eagle population of that area was sufficient to withstand the mortality occurring within it.

Note that this approach to estimating population cost is not limited to windfarms and other spatially localized hazards, but can apply to a variety of mortality regimes so long as an expected annual number of fatalities can be estimated. Our method does, however, require knowledge of background population vital rates, and the latter are difficult to obtain with precision. With regard to the applicability of our analysis to the current effect of the Altamont windfarm on the golden eagle population, we acknowledge that much of the data we draw on is relatively old, and that conditions have changed with recent repowering efforts [66]. Moreover, our 5-year sample of reproduction surveys was doubtless insufficient to accommodate weather effects, including the periodicity of drought cycles in this region [22] and predictions thereof [67]. Another contingency was that reproductive performance in the core study area may not have accurately represented that of the entire DR study area with its greater array of habitat variation [22]. These and other uncertainties suggest that the value of the analysis lies mainly in what it reveals about the proportional cost of human-related mortality to raptor populations, particularly those species with delayed maturity and naturally low reproductive rates. Even so, and despite the many challenges associated with this kind of approach, there will be cases in which the impact of a mortality agent must be evaluated in the absence of vital rates data specific to an area. Here, the application of general estimates might nevertheless yield useful approximations of the burden upon a population of a given number of fatalities within the context of its metapopulation.

## Supporting information

S1 Appendix Field techniques. (PDF)

S2 Appendix Population growth model. (PDF)

S3 Appendix Estimating the floater-to-breeder ratio. (PDF)

S4 Appendix Estimating the demographic cost of windfarm fatalities. (PDF)

S5 Appendix Proportional occurrence of eagle life-stages in the windfarm. (PDF)

S6 Appendix Known fate model selection results. (PDF)

S1 Dataset Known fate capture histories. (XLSX)
