# Supplementary material for: Quantifying the demographic cost of human-related mortality to a raptor population
Source: PLoS One. 2017 Feb 24;12(2):e0172232. doi: 10.1371/journal.pone.0172232 (PMC5325282; doi:10.1371/journal.pone.0172232)
Supplement: S1 Appendix — (PDF) [file pone.0172232.s001.pdf]

S1 Appendix. Field Techniques

Nesting Territory Occupancy and Reproduction

We began surveying eagle territories in January and early February when pairs were conspicuously engaged in undulation displays prior to egg laying (Harmata 1982, Wiens et al. 2015). We chose ground observation points affording clear views of focal-use areas of territorial pairs, yet sufficiently distant that the behavior of the birds appeared (to us) unaffected. Evidence of territory occupancy included observations of pair members perching together, copulating, building or repairing nests, undulating, attacking intruders, carrying prey, soaring together, and vocalizing to one another (Kochert et al. 2002). We returned to territories to determine whether broods were present, and once more to count and age fledged young. Identifying subadults as pair members involved an appraisal of tail plumage. Most adults were readily identifiable as having no distinct white patches in the tail, although a few showed atypical pale areas requiring additional scrutiny.

Capture and Radio-tagging

We captured free-ranging eagles by means of radio-controlled bow-nets and power snares (Jackman et al. 1994), or pit traps (Bloom et al. 2007). We hooded each captured eagle to avoid stress, and attached 65-g VFH transmitters (Biotrack Ltd.) in backpack configuration using 1.3-mm Teflon ribbon held together with cotton embroidery thread over the carina, a procedure designed to allow the transmitter to eventually fall off (Hunt et al. 1992). Transmitters weighed 1.3% of the average weight of females and 1.7% of males. Transmitters were 7 cm long, 3 cm wide, and 2 cm thick, covered with epoxy resin, gently rounded in shape, and dark brown in color; their antennas were 13 cm long and coated with black plastic. Each unit was equipped with a mortality sensor designed to activate when motionless for four hours. The manufacturer estimated battery life at 4 years. As an example of transmitter endurance, the transmitters of 28 breeders (that outlived their transmitters) had median lives of 45 months (SD = 15.6, range = 14-76 months).

Aging and Sexing

We aged nestling golden eagles according to plumage characteristics described by Hoechlin (1976), and older individuals according to Bloom and Clark (2007). We sexed captured free-ranging eagles and 7─9-week-old fledglings by measuring tarsus widths (lateral and dorsoventral), hallux, culmen, beak depth, mass, central rectrix, eighth primary, and wing chord **(Bortolotti 1984). The only non-overlapping measurement for gender identification among the** fledglings (N =126) and among adult and subadult eagles (N =165) was lateral tarsus width, as verified by DNA polymerase chain reaction gender analysis (ZooGen, Inc.) of 20 fledglings and 14 free-ranging golden eagles.

References

Bloom PH, Clark WS, Kidd JW. Capture techniques. In: Bird DM, Bildstein KL, editors. Raptor research and management techniques. Blaine: Hancock House; 2007. pp. 193-219.

Bloom PH, Clark WS. 2001. Molt and sequence of plumages of golden eagles and a technique for in-hand ageing. N Am Bird Band. 2001;26: 97-116.

Bortolotti GR. 1984. Age and sex size variation in golden eagles. J Field Ornithol. 1984;55: 54-66.

Harmata AR. What is the function of undulating flight display in golden eagles? J Raptor Res. 1982;16: 103-109.

Hoechlin DR. Development of golden eaglets in southern California. West Birds. 1976;7: 137-152.

Hunt WG, Jenkins JM, Jackman RE, Thelander CG, Gerstell AT. Foraging ecology of bald eagles on a regulated river. J Raptor Res. 1992;26(4): 243-256.

Jackman RE, Hunt WG, Driscoll DE, Lapsansky F. Refinements to selective capture techniques: a radio-controlled bow net and power snare for bald and golden eagles. J Raptor Res. 1994;28(4): 268-273.

Kochert MN, Steenhof K, McIntyre CL, Craig EH. Golden eagle (*Aquila chrysaetos*). In: Poole A, editor. The birds of North America. Ithaca: Cornell Lab of Ornithology; 2002. Available: http://bna.birds.cornell.edu/bna/species/684.

Wiens JD, Kolar PS, Fuller MR, Hunt WG, Hunt TL. Estimation of occupancy, breeding success, and predicted abundance of golden eagles (*Aquila chrysaetos*) in the Diablo Range, California, 2014. U.S. Geological Survey. 2015. Report No.: 2015-1039. Available: http://dx.doi.org/10.3133/ofr20151039.
