# Supplementary material for: Quantifying the demographic cost of human-related mortality to a raptor population
Source: PLoS One. 2017 Feb 24;12(2):e0172232. doi: 10.1371/journal.pone.0172232 (PMC5325282; doi:10.1371/journal.pone.0172232)
Supplement: S2 Appendix — (PDF) [file pone.0172232.s002.pdf]

S2 Appendix. Population Growth Model

We will refer to the demographic models constructed in Appendices 2 – 4 as post-reproductive pulse models, i.e., the population stages are censused immediately after the ‘reproductive’ pulse. For our applications, the pulse is the annual fledging event since fecundity was measured in terms of fledglings and all eagles in our study were aged from fledging. Breeders are therefore modelled as surviving from one fledging event to the next in order to reproduce successfully (i.e., a breeder that dies between the laying of eggs and the fledging pulse is assumed not to fledge its offspring). But our models are easily adapted to the more common birth-pulse demography if that proves desirable for other applications. All ages are with respect to the reproductive event that spawned the individual in question.

For the computation of λ_p we employed a single-sex (female), reproductive-pulse matrix model with a time step of 12 months. As the population is censused immediately after ‘reproduction,’ the youngest age class included in the census is ‘0-year olds,’ fledglings in our case. With $N(t)$, $J(t)$, $S_1(t)$, $S_2(t)$, and $B(t)$ the number of fledglings, ‘juveniles’ (12 months old), 24- and 36-month-old subadults (at 48 months, surviving subadults transition to the breeder stage), and breeders in year t, respectively, and with f the birth rate, and $j,s,\alpha,$ the juvenile, subadult, and breeder survival rates, respectively, one has

$$\begin{aligned}
N(t+1) &= fB(t+1) = f\alpha B(t) \\
J(t+1) &= N(t)\,j \\
S_1(t+1) &= J(t)s \qquad\qquad (S2.1) \\
S_2(t+1) &= S_1(t)s \\
B(t+1) &= S_2(t)s + B(t)\alpha
\end{aligned}$$

and so the matrix model is

$$\begin{pmatrix} 0 & 0 & 0 & 0 & f\alpha \\ j & 0 & 0 & 0 & 0 \\ 0 & s & 0 & 0 & 0 \\ 0 & 0 & s & 0 & 0 \\ 0 & 0 & 0 & s & \alpha \end{pmatrix}. \qquad (S2.2)$$

Note that in this model we assume subadults cannot successfully lay eggs at any age and that the youngest age at which individuals will successfully fledge offspring is 60 months.

The eigenvalue equation for the matrix (S2.2) is

$$\lambda^5 - \alpha\lambda^4 - fjs^3\alpha = 0 \qquad (S2.3)$$

Hence, the value of λ_p for this model is the largest solution of this equation, which was solved using function 'eigen' in program R.

In order to compute the variance of λ_p by the delta method, one requires the partial derivatives of λ with respect to each of the other parameters. These partial derivatives may be computed easily by implicit differentiation of equation (S2.3). One obtains:

$$\frac{\partial\lambda}{\partial f} = \frac{js^3\alpha}{\lambda^3(5\lambda - 4\alpha)} \qquad \text{(S2.4)}$$

$$\frac{\partial\lambda}{\partial j} = \frac{fs^3\alpha}{\lambda^3(5\lambda - 4\alpha)} \qquad \text{(S2.5)}$$

$$\frac{\partial\lambda}{\partial s} = \frac{3fjs^2\alpha}{\lambda^3(5\lambda - 4\alpha)} \qquad \text{(S2.6)}$$

$$\frac{\partial\lambda}{\partial \alpha} = \frac{\lambda^4 + fjs^3}{\lambda^3(5\lambda - 4\alpha)} \qquad \text{(S2.7)}$$

The variance-covariance matrix is diagonal since the parameters are independent:

$$\Sigma = diag(Var(f), Var(j), Var(s), Var(\alpha)).$$

If $V = (\frac{\partial\lambda}{\partial f}, \frac{\partial\lambda}{\partial j}, \frac{\partial\lambda}{\partial s}, \frac{\partial\lambda}{\partial \alpha})$ the delta method asserts that

$$Var(\lambda) \approx V\Sigma V^T,$$

where V^T denotes the transpose of the row vector V. Consequently,

$$Var(\lambda) \approx Var(f)\left(\frac{\partial\lambda}{\partial f}\right)^2 + Var(j)\left(\frac{\partial\lambda}{\partial j}\right)^2 + Var(s)\left(\frac{\partial\lambda}{\partial s}\right)^2 + Var(\alpha)\left(\frac{\partial\lambda}{\partial \alpha}\right)^2 \qquad \text{(S2.8)}$$

An alternative model is to assume that individuals aged 48 months at a reproductive pulse are treated as adult and as having participated in that reproductive event. For our study, in which the reproductive pulse is fledging, that means these individuals laid eggs at the previous egg laying event when they were, strictly speaking, still subadults. For golden eagles, there is evidence for this behavior, so we consider this model too. The assignment of the adult fecundity rate to these individuals, however, reflects ignorance on our part rather than knowledge. In this alternative, replace the zero term in the first row and penultimate column of the matrix (AS2.2) by *fs*, which is equivalent to adding a term fsS_2 to the right-hand side of the first equation in (S2.1). (S2.3) is then replaced by

$$-\lambda(\lambda^4 - \alpha\lambda^3 - jfs^3) = 0 \qquad \text{(S2.93)}$$

and so λ_p now solves

$$\lambda^4 - \alpha\lambda^3 - jfs^3 = 0\,. \qquad \text{(S2.10)}$$

Equations (S2.4 – 7) are replaced by

$$\frac{\partial\lambda}{\partial f} = \frac{js^3}{\lambda^2(4\lambda - 3\alpha)} \qquad \text{(S2.11)}$$

$$\frac{\partial\lambda}{\partial j} = \frac{fs^3}{\lambda^2(4\lambda - 3\alpha)} \qquad \text{(S2.12)}$$

$$\frac{\partial\lambda}{\partial s} = \frac{3\,fjs^2}{\lambda^2(4\lambda - 3\alpha)} \qquad \text{(S2.13)}$$

$$\frac{\partial\lambda}{\partial \alpha} = \frac{\lambda}{4\lambda - 3\alpha}, \qquad \text{(S2.14)}$$

respectively. *Var*(λ) is then computed as in (S2.8)).

Note that as the main impact of human-induced mortality, including turbine strikes, in the potential growth model is on subadults rather than breeders, and since λ is most sensitive to breeder survival, the improvement in λ after censoring human-induced mortality is somewhat moderate. The potential growth model does not account for the fact that human-induced mortality of floaters is greater than is human-induced mortality of breeders, i.e., the potential growth models with any human-induced mortality underestimate the full effect of that mortality by not including any of its effects on adults. Thus, the potential growth of the population with all human-induced mortality censored is actually a greater improvement over the scenario with human-induced mortality than the modelling indicates.
