# Supplementary material for: Quantifying the demographic cost of human-related mortality to a raptor population
Source: PLoS One. 2017 Feb 24;12(2):e0172232. doi: 10.1371/journal.pone.0172232 (PMC5325282; doi:10.1371/journal.pone.0172232)
Supplement: S3 Appendix — (PDF) [file pone.0172232.s003.pdf]

S3 Appendix. Estimating the Floater-to-Breeder Ratio

We modified the matrix model of Appendix S2 to include floaters as a stage as follows. Stages consisted of fledglings $N(t)$, juveniles $J(t)$, subadults aged two years $S_1(t)$, subadults aged three years $S_2(t)$, and breeders $B(t)$ as in Appendix S2. Subadults aged three years transitioned to floaters over one time step by surviving that year at the subadult survival rate. Floaters had a known-fates survival rate ϕ (floaters that transitioned to breeder status during the year were censored for that estimation of ϕ). A floater that survives the year has two possible fates: it survives for the fraction u of the year as a floater, then either remains a floater with probability (1 – τ), or transitions to a breeder. The transition rates for these two possibilities are $\phi^u(1-\tau)\varphi^{1-u} = \phi(1-\tau)$ and $\phi^u\tau\alpha^{1-u}$, respectively. If there is a particular time of year when floater-to-breeder transition is most likely to occur, that time would determine the value of u. If, on the other hand, any time is equally likely, then the mean of $\phi^u\tau\alpha^{1-u}$ is given by

$$\int_0^1 \phi^u\tau\alpha^{1-u}du = \tau\alpha\int_0^1\left(\frac{\phi}{\alpha}\right)^u du = \tau\frac{\phi-\alpha}{\ln(\phi)-\ln(\varepsilon)} =: \tau\chi\,. \tag{S3.1}$$

Similarly, breeders that survive the year have two fates: they survive as breeders with transition rate (1-σ)α or end the year as floaters, with transition rate $\alpha^u\sigma\phi^{1-u}$ if transition occurs a fraction u into the year and $\sigma\frac{\alpha-\phi}{\ln(\alpha)-\ln(\phi)} = \sigma\chi$, if one takes the mean over all times of transition within the year. In general, denote the floater-to-breeder transition rate by $\chi_1\tau$ and the breeder-to-floater transition rate by $\chi_2\sigma$. With other parameters as in Appendix 1, the matrix model is

$$\begin{pmatrix} 0 & 0 & 0 & 0 & f\chi_1\tau & f\alpha(1-\sigma) \\ j & 0 & 0 & 0 & 0 & 0 \\ 0 & s & 0 & 0 & 0 & 0 \\ 0 & 0 & s & 0 & 0 & 0 \\ 0 & 0 & 0 & s & \phi(1-\tau) & \chi_2\sigma \\ 0 & 0 & 0 & 0 & \chi_1\tau & \alpha(1-\sigma) \end{pmatrix} \tag{S3.2}$$

This model describes an equilibrium with floaters (i.e., precludes subadults from transitioning directly to breeders) which is the situation we are interested in. An equilibrium without floaters, in which subadults transition directly to the breeding stage, can also be modeled, but is not our concern here. At equilibrium, one therefore has the equations:

$$\begin{aligned} &f\chi_1\tau F + f\alpha(1-\sigma)B = N \\ &jN = J \\ &sJ = S_1 \\ &sS_1 = S_2 \\ &sS_2 + \phi\,(1-\tau)F + \chi_2\sigma B = F \\ &\chi_1\,\tau F + \alpha(1-\sigma)B = B \end{aligned} \tag{S3.3}$$

Rewriting the final equation as $\chi_1\, \tau F = [1 - \alpha(1 - \sigma)]B$, permits one to substitute for $\chi_1\tau F$ in the first equation and for τF in the penultimate equation. The first substitution yields $N = fB$, and then successive substitution with this and the second through fourth equations yields $S_2 = fjs^2B$. Substituting in the fifth equation, after the elimination of the τF term, then yields, after straightforward rearrangement,

$$\frac{F}{B} = \frac{\chi_1 fjs^3 + \chi_1\chi_2\sigma - \phi[(1-\alpha(1-\sigma)]}{\chi_1(1-\phi)}. \qquad \text{(S3.4)}$$

For the study population, only one tagged breeder converted to become a floater. If one takes $\sigma = 0$, one has

$$\frac{F}{B} = \frac{\chi_1 fjs^3 - \phi(1-\alpha)}{\chi_1(1-\phi)}. \qquad \text{(S3.5)}$$

Note that the stable stage distribution, at equilibrium, expressing life stages as multiples of the number of breeders is readily obtained from (S3.4) or (S3.5) and (S3.3). For a population in which breeder-to-floater transitions are an important component of the life cycle, however, the dependence of (S3.4) on σ is of interest. Rewrite (S3.4) as

$$\frac{F}{B} = \frac{\chi_1\chi_2 - \phi\alpha}{\chi_1(1-\phi)}\sigma + \frac{\chi_1 fjs^3 - \phi(1-\alpha)}{\chi_1(1-\phi)}. \qquad \text{(S3.6)}$$

If there is a most likely time in the year when floater-breeder transitions occur, it would presumably be due to displacement of breeders by floaters, so breeders that survive such displacement would transition to floaters at the same time as floaters transition to breeders and $\chi_1 = \phi^u\alpha^{1-u}$ and $\chi_2 = \alpha^u\phi^{1-u}$, whence $\chi_1\chi_2 = \alpha\phi$ and the coefficient of σ in (S3.6) is zero, yielding the same result as (S3.5) for the floater-to-breeder ratio at equilibrium.

If instead, one determines χ_1 and χ_2 by mean values as in (S3.1), then

$$\chi_1 = \chi_2 = \frac{\alpha - \phi}{\ln(\alpha) - \ln(\phi)} = \frac{\alpha - \phi}{\ln(\alpha/\phi)} \qquad \text{(S3.7)}$$

Now the response of F/B to σ in (S3.6) is linear, and increases or decreases with increasing σ, according as the sign of $\chi_1\chi_2 - \phi\alpha$ is positive or negative, respectively. Under (S3.7), this quantity is $\chi^2 - \phi\alpha$. Writing $\alpha = \phi(1+x)$, then $\alpha/\phi = 1 + x$, and for small x one can expand $\ln(\alpha/\phi)$ in the power series $\sum_{k=1}^{\infty}(-1)^{n-1}x^n/n$. Using this expression, one can derive the approximation

$$\chi^2 - \alpha\phi \approx \phi^2 \frac{x^2 - x^3}{12},$$

which for x small in magnitude is positive, i.e., under (S3.7) the coefficient of σ in (S3.6) is positive when the difference between α and ϕ is a fraction of ϕ. Hence, in these circumstances, F/B increases with increasing σ, though the coefficient of σ is very small so the effect is very small. The quadratic approximation to χ is $\chi \approx \phi(1 + x/2 - x^2/12)$ so $\phi < \chi < \alpha$. Thus, under (S3.7), (S3.6) includes a very small positive contribution over and above (S3.5). Using the survival rates with human-induced mortality censored (Table 4), the coefficient of σ is 0.00014.

For our purposes at least, (S3.5) serves to estimate the floater-to-breeder ratio at equilibrium. It therefore remains to choose a value of χ_1. We chose two values:

$$\chi_1 = \phi^{1/2}\alpha^{1/2} \qquad \text{(S3.8)}$$

and (S3.7). For all three sets of survival rates, the geometric mean (S3.8) coincided with (S3.7) to three decimal places, whence it sufficed to report a single floater-to-breeder ratio per data set.
