# Supplementary material for: Quantifying the demographic cost of human-related mortality to a raptor population
Source: PLoS One. 2017 Feb 24;12(2):e0172232. doi: 10.1371/journal.pone.0172232 (PMC5325282; doi:10.1371/journal.pone.0172232)
Supplement: S4 Appendix — (PDF) [file pone.0172232.s004.pdf]

S4 Appendix. Estimating the demographic cost of windfarm fatalities

We quantify the demographic cost of the wind turbine-induced mortality as follows. With background vital rates that do not include blade-strike deaths, imagine a population of territorial raptors in which each annual cohort contains just enough survivors at the age of first reproduction to exactly fill the territorial vacancies that accrue during the course of a year as a result of breeder mortality. The population is thus at equilibrium, albeit an idealized one that is nevertheless useful for our purposes. At each reproductive pulse, the population consists only of adult pairs occupying territories and a contingent of free-ranging juveniles and subadults. Each cohort undergoes attrition as a result of turbine deaths and other forms of (background) mortality, and the number of breeding pairs is exactly stabilized by what remains of the cohort when its members reach the age of first reproduction. The number of territories in this idealized equilibrium is our proposed measure of the demographic cost of the blade-strike fatalities. In our models, this number turns out to be linearly proportional to the annual number of blade-strikes and thus can be standardized as the demographic cost of a single annual blade-strike death.

Consider then a population of B territories occupied by breeding pairs for which the background survival rates are j, s, ϕ, and α for juveniles, subadults, floaters, and breeders, respectively, i.e., these survival rates do not include any turbine-induced deaths. Also, let b be the fecundity per breeding pair (at fledging for our study), which is the rate of production of both male and female fledglings (0.64 for this study). For generality, suppose subadults become adults after q years as a subadult (q = 3 in this study). Now individuals aged q+1 may or may not be ready to reproduce at the reproductive pulse coinciding with that age. If not, they must produce the survivors who at the next reproductive pulse are mature enough to fill breeder vacancies and reproduce at the normal rate. Let k denote the rate of survival of individuals aged q+1 a further year; we assume one further year suffices for reproductive maturity. If in fact individuals of age q+1 are ready to breed, one can simply take k = 1. On the other hand, suppose that on average, individuals require a further fraction p of a year before they actually transition from floater to breeder. Then $k = \phi^p \alpha^{1-p}$.

After a reproductive pulse, $(1 - \alpha)B$ breeders die during the following year. By the time of the next reproductive pulse, these vacancies must be exactly filled by the surviving members of that cohort whose members are just old enough to reproduce at that reproductive pulse.

Model 1. In the text, a method for computing the number B of territories in our idealized equilibrium, given the mean age A of blade-strike death (40 months in our study) and assigning all turbine deaths to that age. This model is useful when a complete distribution of the ages of death by turbine is difficult to determine, whereas an estimate of the mean is available from a sample. We first formalize the outline provided in the text. Suppose the mean age A (measured in months) is that of a subadult (as in our study). The number of individuals from B_0 territories surviving to that age, taking into account only the background mortality, is

$$B_0 bj\, s^{(A-12)/12}. \qquad \text{(S4.1)}$$

Setting this expression equal to P models the situation in which the entire cohort that survives to age A months is then extinguished by the annual turbine-induced mortality. Thus,

$$B_0 = \frac{P}{bjs^{(A-12)/12}}\,. \qquad \text{(S4.2)}$$

None of this cohort is left to replace breeders that die (from background mortality as the turbine-induced mortality is already accounted for). Thus, B_0 is an initial estimate of the number B of territories required to *sustain* the annual turbine-induced mortality. The per-territorial-pair production that survives to the age of their first reproduction is $bjs^q k$. Each year, $2B_0(1 - \alpha)$ of the $2B_0$ breeders die and must be replaced, so one needs an additional B_1 territorial pairs, where

$$B_1 bjs^q k = 2B_0(1 - \alpha) \qquad \text{(S4.3)}$$

But $2B_1(1 - \alpha)$ of these die each year as breeders and must be replaced, requiring a further B_2 territorial pairs, given by

$$B_2 bjs^q k = 2B_1(1 - \alpha) \qquad \text{(S4.4)}$$

and so on, ad infinitum, though in practice one soon reaches a number B_i that is less than one and so can be ignored. However, an algebraically elegant result follows by summing to infinity. So, the total number of territorial pairs required is

$$\sum_{i=0}^{\infty} B_i \;=\; B_0 + B_1 + B_2 + \ldots \;=\; B_0 + \frac{2B_0(1-\alpha)}{bjs^q k} + \frac{2B_1(1-\alpha)}{bjs^q k} + \ldots$$

$$= B_0 + \frac{2(1-\alpha)}{bjs^q k}\left[B_0 + B_1 + B_2 + \ldots\right]$$

$$= B_0 + \frac{2(1-\alpha)}{bjs^q k}\sum_{i=0}^{\infty} B_i$$

Solving this equation algebraically for $\sum_{i=0}^{\infty} B_i$ yields (note that (S4.2) is used to substitute for B_0 in the numerator in the following expression):

$$\sum_{i=0}^{\infty} B_i = \frac{B_0}{1 - \dfrac{2(1-\alpha)}{bjs^q k}} = \frac{B_0 bjs^q k}{bjs^q k - 2(1-\alpha)} = \frac{Pbjs^q k \big/ bjs^{(A-12)/12}}{bjs^q k - 2(1-\alpha)} = \frac{Pks^{q-(A-12)/12}}{bjs^q k - 2(1-\alpha)]}\,. \qquad \text{(S4.5)}$$

Now, q is the number of years of subadulthood; measured in months, it can be written as say $(D - 12)/12$, where D is the age in months upon reaching adulthood. Hence, $A < D$, and

$$s^{q-(A-12)/12} = s^{(D-A)/12} \qquad \text{(S4.6)}$$

Thus, by substituting (S4.6) into (S4.5), one obtains

$$B = \frac{Pks^{(D-A)/12}}{bjs^q k - 2(1-\alpha)}, \qquad \text{(S4.7)}$$

as the number of territories required in Model 1. As promised, the result depends linearly on P. This is the result that the stepwise summation outlined in the text converges to.

Note that (S4.7) can be obtained directly by following the trajectory of a cohort through time to find the number that will just fill the $2(1 - \alpha)B$ vacancies at the right time. Indeed, after attrition with respect to background mortality, the survivors to age A of a cohort from the B territories is $Bbjs^{(A\text{-}12)/12}$. Of these, P die from blade-strikes at that age, leaving $Bbjs^{(A\text{-}12)/12} - P$ survivors. These individuals experience further attrition due to background mortality, resulting in $(Bbjs^{(A\text{-}12)/12} - P)s^{(D\text{-}A)/12}k$ survivors at their age of first reproduction. Our idealized equilibrium is characterized by

$$(Bbjs^{(A\text{-}12)/12} - P)s^{(D\text{-}A)/12}k = 2(1\text{-}\alpha)B. \qquad \text{(S4.8)}$$

Solving (S4.8) for B yields exactly (S4.7).

Note that from (S4.7) it is clear that B increases with increasing A (which ranges from 0 to D).

Model 2. Suppose now the age distribution of blade-strike deaths is known. For generality, we now make assumptions about this age distribution; for example, juvenile ages are not excluded. Once again, the model follows a cohort, accounting for attrition from background mortality and turbine-induced mortality according to the age distribution of blade-strike deaths, yielding a generalization of the argument leading to (S4.8). Let x be the first age at which turbine deaths occur and suppose the number P_1. The B territories in the idealized equilibrium produce a cohort of Bb fledglings at each reproductive pulse. For the sake of argument, suppose $x < 12$, i.e., the first turbine casualty is a juvenile. Of the initial cohort, $Bbj^{x/12}$ remain alive at age x after background attrition, and of these, P_1 die from turbines, leaving $Bbj^{x/12} - P_1$. Let the next age at which individuals die from turbines, say P_2 in number, be y, and for sake of argument suppose it is a subadult age, i.e., $y > 12$. After background attrition, there are $(Bbj^{x/12} - P_1)j^{(12\text{-}x)/12}s^{(y\text{-}12)/12}$ survivors, whence $(Bbj^{x/12} - P_1)j^{(12\text{-}x)/12}s^{(y\text{-}12)/12} - P_2$ remain after the turbine-induced mortality at age y. If the next age at which individuals die from turbines, say P_2 in number, is z, another subadult age, then after background attrition and turbine-induced mortality, there remain alive

$$[(Bbj^{x/12} - P_1)j^{(12\text{-}x)/12}s^{(y\text{-}12)/12} - P_2]s^{(z\text{-}y)/12} - P_3. \qquad \text{(S4.9)}$$

Continue in this fashion through the complete age distribution until the oldest age w. Assume that age is at most the age at which individuals reach adulthood (the alternative is elaborated on below). If this age is greater than q+1, then floaters in the population from which the age distribution has been extracted are being killed by turbines. In the idealized equilibrium population, the only floaters are those that survive for at most one year, from age q+1 to either death (natural or otherwise) or to fill a vacancy at their first age of reproduction. Thus, in our model, the oldest age in the distribution of turbine-induced deaths must be less than q+2. Actual deaths at older ages must be reassigned to the interval (q+1,q+2) for the model. The exact form of the survival rate t subject to background attrition from the penultimate age of turbine-induced death to w will depend on w and may involve both the subadult and floater survival rates. After all turbine-induced deaths are accounted for, one has an expression of the form

$$[\ldots[[(Bbj^{x/12} - P_1)j^{(12\text{-}x)/12}s^{(y\text{-}12)/12} - P_2]s^{(z\text{-}y)/12} - P_3]\ldots]t - P_n$$

where P_n is the number of turbine-induced deaths at age w. Finally, let h be the background survival rate from age w to the age of first reproduction. Again, the exact form of h will depend on w, and may involve subadult, floater, and breeder survival rates (in the same way that k above did). Hence, our idealized equilibrium will be characterized by

$$[[\ldots[[(Bbj^{x/12} - P_1)j^{(12-x)/12}s^{(y-12)/12} - P_2]s^{(z-y)/12} - P_3]\ldots]t - P_n]h = 2(1-\alpha)B \qquad \text{(S4.10)}.$$

This equation can be solved for B, being just a somewhat more complicated expression than (S4.8).

We assumed that no breeders were struck by turbine blades, a somewhat typical condition at the Altamont windfarm, where breeders do not usually enter the windfarm. If one needs to allow for turbine-induced breeder deaths, however, note that such deaths create territory vacancies and must be treated differently from blade-strike deaths of juveniles, subadults, and adults that have not yet bred. The age of breeders killed by turbines would be irrelevant; these deaths create additional vacancies that must be filled to maintain territory occupancy and would add an additional term to the right-hand side of (S4.10).

In the absence of any information about the age distribution of blade-strike deaths other than information that restricts such deaths to specific stages, one could assume deaths are evenly distributed across all ages within those life-stages. For example, if turbine-induced deaths are known to be restricted (predominantly) to subadults, one could use (S4.7) with A being the mean subadult age. For our study that would be 30 months, and would therefore provide an underestimate of the demographic cost.

Note that neither Model 1 nor Model 2 is spatially explicit; neither model says anything about the distribution of territories in relation to the spatially localized hazard that the windfarm poses, nor which members of a cohort are at risk. The idealized model merely computes the minimal number of territories needed to sustain a given annual turbine-induced mortality count. A spatially explicit model of which territories produce offspring at risk to any spatially localized hazard is another matter and would be population and hazard specific.
