# Supplementary material for: Quantifying the demographic cost of human-related mortality to a raptor population
Source: PLoS One. 2017 Feb 24;12(2):e0172232. doi: 10.1371/journal.pone.0172232 (PMC5325282; doi:10.1371/journal.pone.0172232)
Supplement: S5 Appendix — (PDF) [file pone.0172232.s005.pdf]

S5 Appendix. Proportional occurrence of eagle life-stages in the windfarm

Table S5a. Proportions of individuals detected in the windfarm at least once per month in aerial surveys of 155 subadults, 51 floaters, and 47 breeders. Fatalities in brackets were those encountered after censoring.

	Jun	Jul	Aug	Sep	Oct	Nov	Dec	Jan	Feb	Mar	Apr	May
**Subadult-months in DR study area**	208	202	184	187	135	125	150	145	151	140	153	140
**Proportion in windfarm**	0.36	0.41	0.36	0.34	0.33	0.30	0.21	0.19	0.26	0.35	0.37	0.38
**Turbine blade-strike deaths**	5	1	4	2	1	1	3	-	[1]	1	4	6
**Floater-months in DR study area**	96	89	78	83	63	65	74	75	65	56	64	55
**Proportion in windfarm**	0.25	0.26	0.32	0.34	0.30	0.25	0.22	0.16	0.25	0.30	0.28	0.22
**Turbine blade-strike deaths**	[1]	1	[1]	-	-	[1]	2	-	1	2 [1]	-	[3]
**Breeder-months in DR study area**	123	122	103	123	93	92	119	120	119	90	112	96
**Proportion in windfarm**	0.02	0.05	0.03	0.03	0.03	0.05	0.05	0.03	0.05	0.03	0.02	0.03
**Turbine blade-strike deaths**	-	-	-	-	-	1	-	-	-	-	-	1 [1]

Table S5b. Proportions of individuals detected in the windfarm in at least one aerial survey per month among three (pooled) cohorts of golden eagles radio-tagged as fledglings (1994, 1995, and 1996) and monitored through their third year of subadulthood. Causes of attrition include mortality, dropped transmitters, failed radios (including expired batteries), and possibly emigration. June relocations were omitted because the mid-month transition of age-categories is confusing (see methods). Fatalities in brackets were those encountered after censoring.

	Jul	Aug	Sep	Oct	Nov	Dec	Jan	Feb	Mar	Apr	May
**Juvenile-months in DR study area**	63	60	59	58	50	48	47	47	48	47	47
**Proportion in windfarm**	0.00	0.00	0.14	0.16	0.24	0.25	0.13	0.40	0.48	0.43	0.51
**Turbine blade-strike fatalities**	-	-	-	-	-	-	-	-	-	-	-
**1st-year subadult-months in DR study area**	44	46	45	41	40	39	37	38	28	34	27
**Proportion in windfarm**	0.55	0.50	0.38	0.44	0.58	0.33	0.35	0.34	0.54	0.56	0.48
**Turbine blade-strike fatalities**	-	-	-	-	-	1	-	-	1	1	1
**2nd-year subadult-months in DR study area**	35	25	28	23	23	30	28	25	18	23	16
**Proportion in windfarm**	0.49	0.48	0.50	0.30	0.43	0.17	0.25	0.44	0.61	0.52	0.75
**Turbine blade-strike fatalities**	-	2	1	-	-	1	-	-	-	1	-
**3rd-year subadult-months in DR study area**	17	12	15	11	9	9	8	4	1	5	1
**Proportion in windfarm**	0.53	0.50	0.60	0.45	0.44	0.11	0.25	0.00	0.00	0.40	1.00
**Turbine blade-strike fatalities**	-	-	-	1	-	1	-	[1]	-	1	[2]
