# Supplementary material for: Quantifying the demographic cost of human-related mortality to a raptor population
Source: PLoS One. 2017 Feb 24;12(2):e0172232. doi: 10.1371/journal.pone.0172232 (PMC5325282; doi:10.1371/journal.pone.0172232)
Supplement: S6 Appendix — (PDF) [file pone.0172232.s006.pdf]

S6 Appendix. Known fate model selection results

Ranking of known-fate models considered for estimation of annual survival for four stage-classes of golden eagles radio-tagged in the vicinity of the Altamont Pass windfarm, 1994–2000. We modeled time effects as constant (.) or varying among seasonal (3-mo) time intervals (*t*). Results shown are based on analyses that included all anthropogenic-caused deaths.

Stage class and model	AIC_c	ΔAIC_c	AIC_c weight	No. parameters	Deviance
Juveniles					
{S(*t*)}	120.860	0.00	0.671	4	3.081
{S(.)}	123.393	2.53	0.189	1	11.727
{S(sex)}	124.846	3.99	0.091	2	11.155
{S(sex**t*)}	126.112	5.25	0.049	8	0.000
Subadults					
{S(.)}	321.172	0.00	0.718	1	26.736
{S(sex)}	323.101	1.93	0.274	2	26.654
{S(*t*)}	330.124	8.95	0.008	12	13.275
{S(sex**t*)}	342.064	20.89	0.000	24	0.000
Floaters					
{S(.)}	117.454	0.00	0.713	1	26.993
{S(sex)}	119.359	1.91	0.275	2	26.873
{S(*t*)}	125.696	8.24	0.012	12	12.247
{S(sex**t*)}	140.449	22.99	0.000	24	0.000
Breeders					
{S(.)}	114.491	0.00	0.732	1	27.404
{S(sex)}	116.506	2.01	0.267	2	27.403
{S(*t*)}	129.775	15.28	0.000	16	11.534
{S(sex**t*)}	153.751	39.26	0.000	32	0.000

AIC_c = Akaike's Information Criterion adjusted for small sample size, ΔAIC_c = difference between the AIC_c value of each model and the lowest AIC_c model, AIC_c wt = Akaike weight.
